# OLDER ADULT PERCEPTIONS OF USING IOT TO SUPPORT MEDICATION ADHERENCE AND COMBAT PHYSICAL INACTIVITY

**DOI:** 10.1093/geroni/igad104.2583

**Published:** 2023-12-21

**Authors:** Yong Kyung Choi, Shih-Yin Lin, Hilaire Thompson, George Demiris

**Affiliations:** University of Pittsburgh, Pittsburgh, Pennsylvania, United States; NYU Rory Meyers College of Nursing, New York City, New York, United States; University of Washington, Seattle, Washington, United States; University of Pennsylvania, Philadelphia, Pennsylvania, United States

## Abstract

The literature has explored the potential of using the Internet of Things (IoT) to support aging in place, but less is known about its ability to motivate older adults for health behavior change. This study aimed to understand older adults’ perception of using IoT to promote medication adherence and combat physical inactivity. We conducted a qualitative secondary analysis of exit interviews of 22 older adults (78% female; mean age: 77.8) who participated in an IoT feasibility evaluation study. In the feasibility study, older adults were asked to choose any combination of the following IoT devices, a door/window sensor, a multi-sensor (motion, light, humidity, temperature), an IP web camera, and a smart speaker, to be installed in their home for 2 months. Thematic analysis revealed that older adults identified creative uses of smart speakers to 1) update, log, and share medication lists/usage and physical activities; 2) set up medication and exercise reminders; 3) support diabetes self-management – taking the guesswork out of the equation (e.g., continuous blood glucose monitoring combined with a smart speaker to recommend the appropriate insulin dosage); 4) read out loud small-print medication labels (when also linked to a smart camera); 5) verbally direct older adults to reach their medications; 6) provide warnings to get up and move when prolonged inactivity is detected by the motion sensor; and 7) bring up music/video for exercise, or serve as a virtual exercise coach. In summary, older adults are receptive to using IoTs to promote medication adherence and reduce physical inactivity.

